# Not enough: Medicaid expansion, medical debt, and cost avoidance in rural American Indian and Alaska Native households

**DOI:** 10.1111/jrh.12925

**Published:** 2025-01-26

**Authors:** Sean Hubbard

**Affiliations:** ^1^ The Center for Health Policy and College of Public Health at The University of North Texas Health Science Center in Fort Worth Fort Worth Texas USA

**Keywords:** Indian Health Service, Medicaid, medical debt

## Abstract

**Purpose:**

Despite expanding health insurance coverage under the Patient Protection and Affordable Care Act (ACA), many Americans struggle with financial barriers to health care. Medicaid expansion was meant to help alleviate these barriers, particularly for rural communities, but has shown mixed results. The American Indian and Alaska Native (AI/AN) community, which faces both racial and geographic disparities, is a group that should benefit from Medicaid expansion. This paper examines differences in medical debt and cost avoidance for rural American Indian and Alaskan Native households in Medicaid expansion and nonexpansion states.

**Methods:**

This study uses data from the National Financial Capability Study in binomial logistic regression models to examine whether AI/AN households in rural areas are more likely to have medical and engage in cost avoidance than their urban counterparts.

**Findings:**

The results show no differences between Medicaid expansion status but do find a higher likelihood of medical debt for those living in rural areas. The results also show a higher likelihood of medical debt and cost avoidance for those living farther from Indian Health Services (IHS)/Tribal health care facilities.

**Discussion/Conclusions:**

The results indicate that rural AI/AN individuals continue to face financial barriers to health care despite the expansion of Medicaid under the ACA. This suggests that rural AI/AN individuals are seeking care outside of the IHS/Tribal system due to limited access to IHS providers. Addressing these barriers may require policies and programs focusing on rural AI/AN communities.

## INTRODUCTION

Despite expanding health insurance coverage under the Patient Protection and Affordable Care Act (ACA), financial barriers remain a defining feature of the US health care system. Many Americans with insurance coverage still struggle with high out‐of‐pocket expenses, copays, and deductibles.^1^ These health‐related expenses leave many who appear to have access to health care facing the choice of whether to take on medical debt or to delay or forgo care.^1^, ^2^ This can mean dire consequences for patients’ physical and financial health, even for those with health insurance.^3^

For those who, by choice or necessity, choose to seek care they cannot afford, this can mean taking on thousands of dollars in medical debt for those with health insurance and hundreds of thousands of dollars for those without coverage.^2^, ^4^, ^5^ Medical debt can be substantially higher in the case of severe or chronic illness, for which treatment often extends more than a single year. This debt can become excessively burdensome for many patients and lead to bankruptcy.^2^, ^5^

Medical debt can have financial consequences beyond bankruptcy. Households who have declared bankruptcy or have high levels of debt are more likely to borrow from predatory lenders such as payday lenders, pawnshops, and auto‐title lenders.^6^, ^7^, ^8^ More specifically, medical debt is associated with higher payday loan debt.^9^ Even more concerning is evidence suggesting that having medical debt is associated with a greater likelihood and a longer length of time that an individual will experience homelessness.^10^

The financial insecurity created by medical debt can have negative consequences on mental and physical health. Evidence suggests that debt level is associated with worse mental health in the form of higher levels of self‐reported stress and depression.^11^ Perhaps related to debt‐induced mental health struggles, debt levels are also associated with worse self‐reported health and higher blood pressure.^12^

Of course, not all patients facing financial barriers take on medical debt. Some, seeking to avoid medical debt, choose to delay or forgo care altogether. Around 25% of Americans report delaying care for severe illness, and 33% report postponing care for any illness due to costs.^13^ Delaying care to avoid the costs of treatment is only a temporary solution and may lead to more serious adverse health outcomes and more expensive treatment.

Engaging in cost avoidance behaviors can often have negative consequences, such as missing early detection of cancers that may be more treatable when detected early but have higher mortality rates and require more expensive treatment when diagnosed at later stages.^14^, ^15^, ^16^, ^17^ Delaying care for chronic diseases such as diabetes, heart disease, and asthma leads to more expensive and invasive treatments and worse outcomes for patients.^18^ Late diagnosis of serious illnesses and unmanaged chronic diseases leads to worse health outcomes and higher costs for both patients and health care systems.^19^

Financial and health consequences of financial barriers are not uniformly distributed. These interrelated impacts can be seen in racial and geographic disparities, with those living in rural areas, African Americans, Latinos, and American Indians/Alaska Natives (AI/AN) being more likely to face health disparities with urban populations and non‐Hispanic Whites.^20^, ^21^, ^22^, ^23^, ^24^, ^25^, ^26^, ^27^, ^28^, ^29^, ^30^ These disparities are most pronounced in AI/AN communities, who, being disproportionately rural, face both racial and geographic disparities.^31^, ^32^, ^33^, ^34^

AI/AN individuals facing disparities may seem counterintuitive, given that many in this community have access to AI/AN‐specific health care options. Members of federally recognized Tribes have access to health care through Indian Health Services (IHS).^35^, ^36^ IHS is responsible for providing and funding direct health care services to eligible members of federally recognized Tribes. IHS‐operated health centers, hospitals, and clinics provide health care for members of 574 federally recognized Tribes in 37 states, totaling 2.5 million American Indian and Alaskan Natives.^37^, ^38^ Access to these programs should insulate AI/AN households from the financial burdens of health care, but this would require the effective delivery of health care through IHS. However, IHS has not sufficiently met these responsibilities due to chronic underfunding and staffing.^39^, ^40^, ^41^

In addition to direct health care services through IHS, members of federally recognized Tribes have access to Tribal ACA Exchanges. Through the Tribal ACA Exchanges, AI/AN individuals are eligible for additional financial assistance, including insurance plans with zero cost‐sharing for those making between 100% and 300% of the federal poverty line (FPL).^42^ This may substitute for Medicaid expansion, but enrollment rates for AI/AN individuals lag behind those of the general population.^42^

In addition to IHS and Tribal ACA Exchanges, members of the AI/AN community have access to the Purchased/Referred Care Program (PRC). The PRC is meant to provide support when American Indians and Alaska Natives seek certain health care services or expertise that IHS cannot provide.^43^ Under PRC, IHS acts as a payer of last resort and pays costs not covered by other sources such as health insurance and Medicaid.^43^ Unfortunately, payment is not guaranteed with PRC, and IHS often lacks the funding to cover the cost of referrals.^44^

These shortcomings are seen in health disparities, such as higher rates of chronic illnesses, substance abuse, and mental health issues, in addition to shorter life expectancy facing AI/AN communities.^31^, ^32^, ^33^, ^34^ These disparities extend into AI/AN households being more likely to have medical debt and to skip filling prescriptions than non‐Hispanic Whites.^45^ This may be related to AI/AN individuals being uninsured at a rate (16%) that is more than twice as high as the uninsured rate for non‐Hispanic Whites (7%).^46^

The ACA does provide a mechanism for addressing these racial and geographic disparities. The law includes a provision expanding Medicaid coverage to include those making less than 138% of the FPL. Evidence suggests that Medicaid expansion may significantly benefit rural populations more than urban ones.^47^ Early results of Medicaid expansion also show significant differences in uninsured rates between AI/AN individuals in expansion and nonexpansion states.^48^

Given the overlap of geographic and racial health disparities facing the AI/AN community and evidence suggesting that Medicaid expansion may help mitigate these disparities, there may be differences in how AI/AN households manage financial barriers to care. This paper examines whether there are differences in medical debt and cost avoidance behaviors by AI/AN individuals living in Medicaid expansion and those living in nonexpansion states in the United States, particularly for those living in rural areas. The results may provide further insights into the benefits of Medicaid expansion rural on AI/AN communities.

## METHODS

This study uses state‐by‐state survey data from the National Financial Capability Study (NFCS) from the Financial Industry Regulatory Authority, Inc. (FINRA) Investor Education Foundation, conducted from June through October 2018. NFCS is an online survey conducted among a nationally representative sample of American adults. Respondents are drawn using nonprobability quota sampling from established online panels of individuals who have agreed to participate in online surveys. Quotas are set for each state to approximate the Census distributions for age by gender, ethnicity, education level, and income.^49^ The data capture perceptions, attitudes, experiences, and behaviors on topics such as personal finances, savings, debt, and financial investment.^49^ More importantly, it includes questions about whether the household has unpaid medical bills, skipped medical treatments, or not filled prescription medications due to costs.

After removing the nonresponses and missing data, a subset was extracted for only those self‐identifying as AI/AN. This resulted in a sample size of 456 individuals. This is a relatively small sample size for a regression model but was larger than the minimum sample size of approximately 311 required for this model.^50^ Characteristics of the study population are shown in Table 1.

**TABLE 1:** Characteristics of study population.

(N = 456)		
	**N**	**%**
**Age**		
18‐29	84	18.42
30‐39	91	19.96
40‐49	79	17.32
50‐64	135	29.61
65 and above	67	14.69
**Educational attainment**		
No high school	14	3.07
High school/GED	87	19.07
Some college	197	43.20
College graduate	106	23.25
Graduate degree	52	11.40
**Married**	203	44.52
**Employment status**		
Not employed	219	48.03
Part‐time	37	8.11
Full‐time	200	43.86
**Annual income**		
Less than $15,000	63	13.82
$15,000‐$24,999	51	11.19
$25,000‐$34,999	74	16.23
$35,000‐49,999	63	13.82
$50,000‐74,999	97	21.27
$75,000‐$99,999	55	12.06
$100,000 and above	53	11.62
**Has health insurance**	387	84.87
**Medicaid expansion**		
Nonexpansion state	183	40.13
Expansion state	273	59.87
**Medicaid/SNAP recipient**	123	26.97

Binomial logistic regression was used to examine whether AI/AN individuals living in Medicaid expansion states are less likely than those in nonexpansion states to have medical debt and engage in cost‐avoidance behaviors: skipped tests, treatments, or recommended follow‐ups, not seeing a doctor when faced with a medical condition, and have not filled prescriptions due to cost.

The model includes a travel distance variable using Euclidean Distance from the center of the participant's ZIP Code to the nearest IHS/Tribal facility.^52^ A variable for rural classification is included using Federal Office of Rural Health Policy Zip Code classifications. Both travel distance and rural classification are included to account for the significant number of IHS/Tribal facilities in rural areas.^52^ Tests revealed no statistically significant correlation between travel distance and rural classification. The models also control for covariates and confounders such as employment status, income, health insurance coverage, and so on.

A cutoff date of 2016 was used to classify states as Medicaid expansion or nonexpansion. Only states that expanded Medicaid coverage before the end of 2016 are included in the Medicaid expansion category, which allows for at least a 2‐year lag in the effects of Medicaid expansion. Twenty‐nine states had expanded Medicaid before the 2016 cutoff date, and 2 states approved but did not implement Medicaid expansion between 2016 and data collection in 2018. The remaining 18 states had not passed Medicaid expansion at the time of data collection. Data cleaning and analysis were conducted in R version 4.4.1 using the tidyverse package.^53^

## RESULTS

The medical debt hypotheses were that AI/AN individuals living in states that expanded Medicaid would be less likely to have medical debt and that there would be no statistically significant difference in the likelihood of AI/AN individuals in rural areas having medical debt. Model 1 examines these hypotheses using the likelihood of a respondent having medical debt. The results are shown in Table 2.

**TABLE 2:** Medical debt results.

	Odds ratio	*P*‐value
**Intercept**	0.35	.095
**Age**		
18‐29	–	–
30‐39	1.23	.602
40‐49	1.39	.439
50‐64	1.07	.869
65 and above	0.65	.392
**Educational attainment**		
No high school	5.53	.015
High school/GED	–	–
Some college	2.38	.015
College grad	1.24	.606
Graduate degree	1.92	.214
**Married**	0.66	.135
**Employment status**		
Not employed	–	–
Part‐time	0.68	.446
Full‐time	0.87	.650
**Annual income**		
< $15,000	–	–
$15,000‐$24,999	2.62	.030
$25,000‐$34,999	0.57	.226
$35,000‐49,999	1.57	.335
$50,000‐74,999	1.07	.881
$75,000‐$99,999	0.51	.266
$100,000 and above	0.26	.043
**Medicaid/SNAP recipient**	1.54	.155
**Insurance**	0.46	.014
**Travel distance**		
0‐25 miles	–	–
26‐50 miles	0.73	.518
51‐75 miles	1.82	.237
76‐100 miles	1.42	.539
101‐125 miles	1.70	.303
126‐150 miles	4.05	.014
151‐175 miles	1.20	.826
176‐200 miles	5.58	.003
201‐250 miles	0.69	.585
251‐300 miles	3.05	.070
More than 300 miles	1.39	.465
**Rural**	1.94	.032
**Medicaid expansion**	0.69	.162
**AIC**	497.78	
**N**	456	

The results show no statistically significant relationship between a state having expanded Medicaid and the likelihood of AI/AN individuals having medical debt. However, there is a correlation between AI/AN individuals living in a rural area and their likelihood of having medical debt. Those living in rural areas are more likely (OR = 1.98) to have unpaid medical bills than those not living in rural areas. Distance from an IHS facility also appears to have a strong relationship with the likelihood of AI/AN individuals having medical debt. Those living a greater distance from an IHS facility, 126‐150 miles (OR = 4.05), 176‐200 miles (OR = 5.58), and 250‐300 miles (OR = 3.05), are more likely to have unpaid medical bills than those who live less than 25 miles from an IHS facility.

The higher likelihood of having medical debt for AI/AN individuals living in rural areas and farther away from IHS facilities suggests that this group is seeking care outside of the IHS system. If rural AI/AN individuals are seeking care outside of the IHS/Tribal system, it implies that they are less likely to engage in cost‐avoidance behaviors. The next models investigate this further by examining 3 cost avoidance behaviors. The results of these models are shown in Table 3.

**TABLE 3:** Cost avoidance behavior results.

	Skipped recommended test, follow‐up, or treatment		Did not fill prescription		Did not see doctor for medical problem	
	Odds ratio	*P*‐value	Odds ratio	*P*‐value	Odds ratio	*P*‐value
**Intercept**	0.11	.002	0.14	.005	0.33	.090
**Age**						
18‐29	–	–	–	–	–	–
30‐39	0.82	.636	1.13	.785	1.34	.464
40‐49	1.03	.938	1.76	.208	1.64	.235
50‐64	1.42	.340	1.57	.263	1.79	.113
65 and above	0.94	.899	0.79	.647	0.89	.819
**Educational attainment**						
No high school	2.67	.1634	4.20	.043	1.25	.747
High school/GED	–	–	–	–	–	–
Some college	2.18	.031	2.04	.060	1.32	.417
College grad	1.41	.400	1.24	.618	1.09	.820
Graduate degree	0.98	.972	1.79	.304	0.57	.291
**Married**	0.98	.950	0.91	.740	1.15	.614
**Employment status**						
Not employed	–	–	–	–	–	–
Part‐time	0.85	.737	0.33	.047	0.59	.289
Full‐time	1.075	.818	0.69	.270	1.12	.707
**Annual income**						
< $15,000	–	–	–	–	–	–
$15,000‐$24,999	3.89	.004	4.82	.001	2.31	.078
$25,000‐$34,999	1.81	.211	2.15	.119	2.55	.041
$35,000‐49,999	2.91	.029	2.76	.045	2.74	.037
$50,000‐74,999	0.85	.748	0.75	.608	0.84	.726
$75,000‐$99,999	0.54	.359	0.95	.935	0.58	.387
$100,000 and above	1.06	.928	0.95	.944	.97	.957
**Has health insurance**	1.06	.161	.36	.005	.26	<.001
**Medicaid/SNAP recipient**	1.22	.522	2.42	.005	1.29	.395
**Travel distance**						
0‐25 miles	–	–	–	–	–	–
26‐50 miles	1.94	.190	1.34	.567	2.736	.033
51‐75 miles	1.21	.747	3.00	.044	2.605	.076
76‐100 miles	2.10	.209	1.81	.325	2.034	.214
101‐125 miles	1.95	.211	0.82	.731	1.584	.382
126‐150 miles	4.33	.014	5.06	.008	4.391	.011
151‐175 miles	1.33	.7754	0.00	.980	.3611	.390
176‐200 miles	4.64	.013	1.95	.517	4.784	.009
201‐250 miles	2.69	.118	2.72	.120	4.770	.010
251‐300 miles	2.58	.161	3.48	.058	1.264	.624
More than 300 miles	1.99	.154	1.47	.430	1.0195	.7896
**Rural**	0.53	.076	0.67	.260	0.47	.032
**Medicaid expansion**	0.90	.687	1.00	.996	0.70	.191
**A1C**	494.04		447.93		505.51	
**N**	456		456		456	

Model 2 examines the likelihood of an individual skipping a recommended test, treatment, or follow‐up appointment due to cost. As with medical debt, there appears to be no statistically significant relationship between AI/AN individuals living in a Medicaid expansion state and the likelihood of skipping a test, treatment, or follow‐up appointment. A relationship does exist between this type of cost avoidance and AI/AN individuals living in rural areas or farther away from IHS facilities.

AI/AN individuals living in rural areas are less likely (OR = 0.53) to skip a recommended test, treatment, or follow‐up than those who do not live in rural areas. The opposite relationship appears concerning distance from an IHS facility. AI/AN individuals who live farther away from an IHS facility, 126‐150 miles (OR = 4.33) and 176‐200 miles (OR = 4.64), are more likely to skip a recommended test, treatment, or follow‐up appointment than those who live less than 25 miles from an IHS facility.

The next type of cost avoidance behavior, not filling a prescription due to costs, shows similar results. There remains no relationship between the behavior and whether an AI/AN household lives in a Medicaid expansion state. Model 3 also reveals no relationship between AI/AN individuals living in a rural area and the likelihood of not filling a prescription. Distance from an IHS facility remains correlated with a higher likelihood of engaging in cost avoidance behavior. AI/AN individuals who live farther from an IHS facility, 126‐150 miles (OR = 5.06) and 251‐300 miles (OR = 3.48), are more likely than those who live less than 25 miles from an IHS facility to skip filling their prescriptions due to cost.

Model 4 examines the final type of cost avoidance behavior: not seeing a doctor when faced with a medical issue. As with the previous models, the results reveal no statistically significant relationship for AI/AN individuals living in a Medicaid expansion state. However, the results do show correlations between the likelihood of AI/AN individuals not seeing a doctor when faced with a medical issue and living in a rural area or farther from an IHS facility.

AI/AN individuals living in rural areas are less likely (OR = 0.47) to report having not seen a doctor when faced with a medical issue than those who do not live in rural areas. As with model 2, distance from an IHS facility shows a different relationship. AI/AN individuals who live farther from an IHS facility, 26‐50 miles (OR = 2.74), 51‐75 miles (OR = 2.61), 126‐150 miles (OR = 4.39), 176‐200 miles (OR = 4.78), and 201‐250 miles (OR = 4.77), are more likely than those who live less than 25 miles from an IHS facility to say that they did not see a doctor when faced with a medical problem.

The results of the cost avoidance models appear to align with the results of the medical debt model. AI/AN individuals living in rural areas are more likely to have debt from unpaid medical bills, which indicates that they do seek medical care, likely outside of the IHS system. Additionally, rural AI/AN individuals are less likely to engage in related cost avoidance behaviors such as skipping recommended tests, treatments, and follow‐ups. This confirms that rural AI/AN individuals are not avoiding medical care due to costs.

## DISCUSSION

These results provide some potentially important insights into financial barriers to care in rural American Indian and Alaskan Native communities. The first is that it appears that both rural and urban AI/AN individuals struggle with financial barriers to health care. The results also suggest that urban and rural AI/AN individuals may have different methods for navigating these barriers. Rural AI/AN individuals are more likely to have medical debt but less likely to engage in cost avoidance than those living in urban areas. This suggests that rural AI/AN individuals are more likely to seek care outside of the IHS/Tribal system than urban AI/AN individuals.

Distance from an IHS/Tribal facility is also an important factor in whether an AI/AN individual has medical debt or engages in cost avoidance. Like rural AI/AN individuals, those living farther from an IHS/Tribal health facility are more likely to have medical debt than those who live less than 25 miles from an IHS/Tribal facility. It also appears that AI/AN individuals living farther from an IHS/Tribal facility are more likely to engage in cost avoidance behavior than those who live less than 25 miles from an IHS facility. The combination of medical debt and cost avoidance behaviors indicates that distance from an IHS facility may present the greatest barrier to care.

Both living in a rural area and living farther from an IHS facility appear to play a more significant role than Medicaid expansion. The analysis found no relationship between Medicaid expansion and cost‐avoidance behaviors among rural AI/AN individuals. This suggests that while Medicaid expansion may have benefits for rural populations generally, it may have little to no effect on the likelihood of AI/AN individuals having medical debt or engaging in cost avoidance. The lack of any impact of Medicaid expansion on medical debt and cost‐avoidance among rural AI/AN individuals implies that Medicaid coverage may be less of an issue for rural AI/AN individuals than access and proximity to IHS/Tribal health care.

## CONCLUSIONS

Even with the expansion of health insurance coverage under the ACA, many vulnerable communities struggle with disparities in access to health care. These disparities are seen in worse physical, mental, and financial health outcomes for non‐Whites and those living in rural areas.^20^, ^21^, ^22^, ^23^, ^24^, ^25^, ^26^, ^27^, ^28^, ^29^, ^30^ These disparities are, at least in part, the result of financial barriers to care, such as copays, deductibles, and out‐of‐pocket maximums.^1^

Due to their disproportionate rural population, AI/AN individuals often face both racial and geographic health disparities.^31^, ^32^, ^33^, ^34^ These disparities exist despite AI/AN‐specific health care systems such as IHS, Purchased and Referred Care, and Tribal ACA exchanges.^39^, ^40^, ^41^, ^43^ While the ACA has had some success increasing insurance coverage in AI/AN communities, evidence of financial barriers remains.^45^, ^46^, ^48^

Understanding the barriers facing rural AI/AN populations is particularly important. While the AI/AN population is increasingly urban, it remains disproportionately rural. AI/AN individuals comprise 2.1% of the rural population but only 0.4% of the urban population in the United States.^54^ This means that geographic disparities and policies may have a greater impact on rural AI/AN individuals.

This paper examined the relationship between ACA Medicaid expansion and evidence of financial barriers facing rural AI/AN individuals. The results find no evidence of a correlation between Medicaid expansion and the likelihood of AI/AN individuals having medical debt or engaging in cost avoidance behaviors. However, they do show evidence that AI/AN individuals in rural areas are more likely to have medical debt and less likely to engage in cost avoidance. The results also show that those living farther from IHS/Tribal facilities are more likely to have medical debt and to engage in cost avoidance than those living less than 25 miles from an IHS/Tribal facility.

This suggests that rural AI/AN individuals and those living farther away from IHS/Tribal facilities are seeking care but doing so outside of the IHS/Tribal system. There are several reasons why AI/AN individuals may be taking on medical debt when seeking care outside of the IHS/Tribal system. One reason is that they may not have obtained coverage through Tribal ACA exchanges and are either uninsured, self‐pay, or have a health insurance plan with high cost‐sharing. Another reason may be that these individuals seek care expecting to be reimbursed under the PRC program, only to find out that the plan is out of money for the year.^44^

Whatever the cause, the presence of medical debt combined with no observed effect of living in a Medicaid expansion state implies that further policy interventions may be needed to address the health care needs of rural AI/AN communities. The relationship between distance from an IHS/Tribal facility and the likelihood of having medical debt and cost avoidance suggests that geographic access may be a barrier for rural AI/AN individuals. If rural AI/AN individuals are accruing medical debt because of a lack of geographic access to health care services, one solution may be to increase the availability of IHS/Tribal providers in rural areas.

An alternative solution may be increased funding for the PRC or more resources for Tribal ACA Exchange promotion. Additional funding for PRC would help ease the financial burden for those AI/AN individuals who are referred to outside providers when the IHS/Tribal system is unable to provide needed care or services. Additional funding to promote Tribal ACA Exchanges may boost enrollment in Tribal health insurance plans and provide coverage for AI/AN individuals to seek care from outside providers when IHS/Tribal facilities are difficult to access. Determining which of these is the correct policy approach or combination of approaches will require further investigation.

These conclusions and policy recommendations should be viewed in the context of this study's limitations. The National Financial Capability Study does not include data on whether AI/AN individuals are members of federally recognized Tribes. This is an important distinction because only members of federally recognized Tribes are eligible for IHS programs and services. Additionally, the dataset does not contain information about the participants’ health conditions or overall health status. Differences in individuals’ health may certainly influence the likelihood that they will have medical debt.

Considering these limitations, future qualitative research should investigate the barriers to care and sources of medical debt for rural AI/AN individuals. Efforts should also be made to account for IHS eligibility by collecting data that identifies members of federally recognized Tribes. The additional information will help inform policy to improve health care access for rural AI/AN communities.

## CONFLICT OF INTEREST STATEMENT

Neither the author nor the author's institution has received any funding or has any conflict of interest related to this manuscript.
